# Supplementary material for: Loop competition and extrusion model predicts CTCF interaction specificity
Source: Nat Commun. 2021 Feb 16;12:1046. doi: 10.1038/s41467-021-21368-0 (PMC7886907; doi:10.1038/s41467-021-21368-0)
Supplement: Supplementary file 2 — Reporting Summary [file 41467_2021_21368_MOESM2_ESM.pdf]

Corresponding author(s): Michael A. Beer

Last updated by author(s): Jan 10, 2021

Reporting Summary

Nature Research wishes to improve the reproducibility of the work that we publish. This form provides structure for consistency and transparency in reporting. For further information on Nature Research policies, see our Editorial Policies and the Editorial Policy Checklist.

Statistics

For all statistical analyses, confirm that the following items are present in the figure legend, table legend, main text, or Methods section.

n/a	Confirmed	
☐	☒	The exact sample size (*n*) for each experimental group/condition, given as a discrete number and unit of measurement
☒	☐	A statement on whether measurements were taken from distinct samples or whether the same sample was measured repeatedly
☒	☐	The statistical test(s) used AND whether they are one- or two-sided<br>*Only common tests should be described solely by name; describe more complex techniques in the Methods section.*
☒	☐	A description of all covariates tested
☒	☐	A description of any assumptions or corrections, such as tests of normality and adjustment for multiple comparisons
☒	☐	A full description of the statistical parameters including central tendency (e.g. means) or other basic estimates (e.g. regression coefficient) AND variation (e.g. standard deviation) or associated estimates of uncertainty (e.g. confidence intervals)
☒	☐	For null hypothesis testing, the test statistic (e.g. F, t, r) with confidence intervals, effect sizes, degrees of freedom and P value noted<br>*Give P values as exact values whenever suitable.*
☒	☐	For Bayesian analysis, information on the choice of priors and Markov chain Monte Carlo settings
☒	☐	For hierarchical and complex designs, identification of the appropriate level for tests and full reporting of outcomes
☒	☐	Estimates of effect sizes (e.g. Cohen's *d*, Pearson's *r*), indicating how they were calculated

Our web collection on statistics for biologists contains articles on many of the points above.

Software and code

Policy information about availability of computer code

Data collection	We used publicly available data, and no additional software was used for data collection.
Data analysis	Our software is available at https://github.com/wangxi001/Loop-Extrusion-Model or http://doi.org/10.5281/zenodo.4404848 as described in the Code Availability section of the manuscript. Other previously published software used (and referenced) and version numbers are: python Xgboost package version 0.90, BWA-MEM version v0.7.8, HICCUPS (juicer tools) v1.13.02, ChIA-PET2 v0.9.3, macs2 v2.2.6, CTCF motif id on Jaspar is MA0139.1

For manuscripts utilizing custom algorithms or software that are central to the research but not yet described in published literature, software must be made available to editors and reviewers. We strongly encourage code deposition in a community repository (e.g. GitHub). See the Nature Research guidelines for submitting code & software for further information.

Data

Policy information about availability of data

All manuscripts must include a data availability statement. This statement should provide the following information, where applicable:

- Accession codes, unique identifiers, or web links for publicly available datasets
- A list of figures that have associated raw data
- A description of any restrictions on data availability

Training data available for download from https://github.com/wangxi001/Loop-Extrusion-Model or or http://doi.org/10.5281/zenodo.4404848. There are no restrictions on its use.

Field-specific reporting

Please select the one below that is the best fit for your research. If you are not sure, read the appropriate sections before making your selection.

☒ Life sciences ☐ Behavioural & social sciences ☐ Ecological, evolutionary & environmental sciences

For a reference copy of the document with all sections, see nature.com/documents/nr-reporting-summary-flat.pdf

Life sciences study design

All studies must disclose on these points even when the disclosure is negative.

Sample size	We used the entire applicable CTCF ChIA-PET dataset for training the model. The sample size of this training set depends on the read-count cutoff for loop determination, which was chosen to be 4 paired-end-tag reads for GM12878 and 3 paired-end-tag reads in Ref. 16.
Data exclusions	All applicable data points were used: all loop anchors with CTCF binding sites were used in the analysis since CTCF binding is a necessary component of our model. Only CTCF ChIA-PET loops which did not have CTCF binding at a loop anchor were excluded.
Replication	We repeated the loop predictions 10 times on 10% sub-samples of the full data to determine the robustness and replicability of the performance metrics, 95% confidence intervals are shown.
Randomization	The only randomization was for cross-fold validation set assignment and was performed randomly.
Blinding	Blinding is not relevant to this computational study, all applicable data points were used (loop anchors with CTCF binding sites).

Reporting for specific materials, systems and methods

We require information from authors about some types of materials, experimental systems and methods used in many studies. Here, indicate whether each material, system or method listed is relevant to your study. If you are not sure if a list item applies to your research, read the appropriate section before selecting a response.

Materials & experimental systems

n/a	Involved in the study	
☒	☐	Antibodies
☒	☐	Eukaryotic cell lines
☒	☐	Palaeontology and archaeology
☒	☐	Animals and other organisms
☒	☐	Human research participants
☒	☐	Clinical data
☒	☐	Dual use research of concern

Methods

n/a	Involved in the study	
☒	☐	ChIP-seq
☒	☐	Flow cytometry
☒	☐	MRI-based neuroimaging
